# Rapid in vitro differentiation of bacteria by ion mobility spectrometry

**DOI:** 10.1007/s00253-021-11315-w

**Published:** 2021-05-11

**Authors:** Isabel Steppert, Jessy Schönfelder, Carolyn Schultz, Dirk Kuhlmeier

**Affiliations:** 1 MicroDiagnostics, Fraunhofer Institute for Cell Therapy and Immunology IZI, Leipzig, Germany; 2 Institute for Biochemistry and Biology, University of Potsdam, Potsdam, Germany; 3 Project Hub Microelectronic and Optical Systems for Biomedicine MEOS, Fraunhofer Institute for Cell Therapy and Immunology IZI, Erfurt, Germany

**Keywords:** Bacteria identification, Volatile organic compounds (VOC), Ion mobility spectrometry (IMS), Antibiotic resistance, Infection, Diagnostic

## Abstract

Rapid screening of infected people plays a crucial role in interrupting infection chains. However, the current methods for identification of bacteria are very tedious and labor intense. Fast on-site screening for pathogens based on volatile organic compounds (VOCs) by ion mobility spectrometry (IMS) could help to differentiate between healthy and potentially infected subjects. As a first step towards this, the feasibility of differentiating between seven different bacteria including resistant strains was assessed using IMS coupled to multicapillary columns (MCC-IMS). The headspace above bacterial cultures was directly drawn and analyzed by MCC-IMS after 90 min of incubation. A cluster analysis software and statistical methods were applied to select discriminative VOC clusters. As a result, 63 VOC clusters were identified, enabling the differentiation between all investigated bacterial strains using canonical discriminant analysis. These 63 clusters were reduced to 7 discriminative VOC clusters by constructing a hierarchical classification tree. Using this tree, all bacteria including resistant strains could be classified with an AUC of 1.0 by receiver-operating characteristic analysis. In conclusion, MCC-IMS is able to differentiate the tested bacterial species, even the non-resistant and their corresponding resistant strains, based on VOC patterns after 90 min of cultivation. Although this result is very promising, in vivo studies need to be performed to investigate if this technology is able to also classify clinical samples. With a short analysis time of 5 min, MCC-IMS is quite attractive for a rapid screening for possible infections in various locations from hospitals to airports.

Key Points

*• Differentiation of bacteria by MCC-IMS is shown after 90-min cultivation.*

*• Non-resistant and resistant strains can be distinguished.*

*• Classification of bacteria is possible based on metabolic features.*

**Supplementary Information:**

The online version contains supplementary material available at 10.1007/s00253-021-11315-w.

## Introduction

Infectious pathogens pose a significant challenge not only to the medical sector but also globally. The worldwide travel rapid global spreading of infectious pathogens within a few days (Olsen et al. 2003; Khan et al. 2009). Besides viruses, spreading of multi-resistant bacteria from one country to another has been reported as well (Molton et al. 2013). In the Netherlands, every admitted foreign patient is screened for multi-resistant bacteria to prevent nosocomial infections (Gunnink et al. 2021). Such control policy has contributed to low incidence of methicillin-resistant *Staphylococcus aureus* (MRSA) infections in Netherlands. Consequently, new rapid tests are extremely sought after to screen for infected subjects on site.

Currently, the standard diagnostic testing of bacteria is based on cultivation and molecular biological methods. Cultivation techniques belong to the core methodology for diagnosis of bacterial infections but it takes days until the results are available (Laupland and Valiquette 2013). To minimize the testing time, genome-based methods like PCR, next-generation-sequencing, and proteome-based matrix-assisted laser desorption ionization-time of flight mass spectrometry (MALDI-TOF-MS) have been developed (van Belkum et al. 2013). Despite the short testing time ranging from 1 h up to 1 day, these tests are still time-consuming and are usually not performed at the point of care. Moreover, these methods are cost-intensive and require many consumables which render them unusable on a grand scale. Consequently, there is an extreme demand for a cost-effective and sensitive analytical device as point of care tool which can detect relevant infections within only some minutes.

On the metabolic level, infectious diseases can be identified based on volatile organic compounds (VOCs) in exhaled breath (Ruszkiewicz et al. 2020; Kunze-Szikszay et al. 2019). VOCs are emitted as gaseous metabolites during the metabolism and provide information about the physiological condition of an organism (Shirasu and Touhara 2011). Recently, many in vitro as well as in vivo studies have proven that different pathogens or diseases result in characteristic combination of VOCs (Hong-Geller and Adikari 2018). Hence, there is a high chance to detect disease-specific VOCs as biomarkers even before the first symptoms occur (Traxler et al. 2018).

Various analytical techniques have been applied for investigating VOCs. The most widely used method is mass spectrometry. Gas chromatography coupled to mass spectrometry (GC-MS) is the gold standard but is quite elaborate in analysis, bulky and requires experts with high expertise (Mathew et al. 2015). In contrast, electronic noses are portable and easy to use. They consist of chemical sensors, which technically imitate the smelling. However, they are limited in quantification precision due to cross reactivity and suffer from sensor ageing (Wilson 2015). Ion mobility spectrometry (IMS) on the other hand has many advantages for diagnostics. It is fast and very sensitive with detection limits of down to parts-per-trillion (ppt) (Westhoff et al. 2009; Hong-Geller and Adikari 2018). In addition, it is already used as on-site tool for the detection of chemical warfare agents, explosives, and drugs at airports (Hopfgartner 2019). Coupling IMS with gas chromatographic columns such as multi-capillary columns (MCC) enhances the separation of complex gas mixtures and provides higher discriminatory power (Cumeras et al. 2015). Due to easy handling and portability, IMS is an ideal candidate for on-site breath analysis. Although laborious, it is even possible to chemically identify the analytes if reference measurements are available. Chemical identification may facilitate a causal interpretation of the results by giving further information about underlying metabolic processes. Nevertheless, pattern recognition may be sufficient for the identification of diseases if the chemical identity of VOCs as biomarkers is known (Westhoff et al. 2009).

While many studies on infection- or pathogen-related VOCs were performed with mass spectrometry technologies, only a few studies have been conducted with ion mobility spectrometry. A differentiation between different bacterial strains and controls in headspace above cultures could be achieved from 2 up to 24 h of incubation (Jünger et al. 2012; Kunze et al. 2013; Steppert 2013; Drees et al. 2019). Slowly growing mycobacteria could be detected with differential ion mobility spectrometry (DMS) after 1 week of cultivation. Compared to that the identification of mycobacteria using classical culturing techniques takes about 6 weeks (Purkhart et al. 2017). In an in vivo study, Sahota et al. revealed that patients with tuberculosis could be differentiated from the healthy control group with a sensitivity and specificity of about 80% based on VOCs in exhaled breath by field asymmetric ion mobility spectrometry (FAIMS) (Sahota et al. 2016).

The goal of our research is to develop an IMS-based method for the rapid identification of infectious pathogens that can be used for on-site breath analysis. As a first step towards this goal, this study investigated bacterial cultures in vitro due to a better reproducibility compared to in vivo tests. Within this study, the general feasibility to differentiate several bacterial strains with MCC-IMS should be proven. Besides five bacterial strains from different genera and families, two resistant strains were included to analyze if these can be differentiated from their corresponding sensitive strain. The successful differentiation of in vitro cultures will allow subsequent studies with more complex clinical samples and breath from infected patients.

## Materials and methods

### Bacterial strains and sample preparation

This study includes five bacterial species which are commonly found in infections. These are *E. coli* (DSM 1576), *S. aureus* (DSM 346), *P. aeruginosa* (DSM 1117), *K. pneumoniae* (DSM 30104), and *A. baumannii* (DSM 30007). For investigating the differences in VOC patterns between non-resistant and resistant bacteria, this study includes two antibiotic-resistant strains, methicillin-resistant *S. aureus* (DSM 13661), and extended-spectrum beta-lactamase (ESBL) producing *K. pneumoniae* (DSM 26371). Both resistant strains are used as reference strains for testing antimicrobial resistance according to Clinical and Laboratory Standards Institute (CLSI), and thus, they were used in this study. Resistance in this MRSA strain DSM 13661 is regulated by the gene mecA located in the staphylococcal cassette chromosome mec (SCCmec) which codes for a modified penicillin-binding protein (PBP2a) with low affinity for beta-lactam antibiotics (Kwiatkowski et al. 2020; Peacock and Paterson 2015).

All bacterial strains were cultivated overnight on tryptic soy agar plates at 37 °C. Afterwards they were sub-cultured in 30-ml tryptic soy broth (TSB) (Oxoid Limited, Hamphire, UK) and incubated for 20 h with constant agitation at 37 °C. On the day of the MCC-IMS measurements, starting cultures with an initial bacterial concentration of 0.1 optical density at 600 nm (OD_600_) were gained from subcultures. An amount of 5 ml of the starting cultures were transferred into 20-ml autoclaved headspace vials (Macherey-Nagel, Düren, Germany) and sealed with autoclaved PTFE/silicone septum caps (Macherey-Nagel, Düren, Germany). Headspace vials with 5-ml pure TSB were prepared as control samples. All filled headspace vials were incubated at 37 °C in a heating block for 90 min until the start of the analysis with MCC-IMS.

To ensure the reproducibility of the method, the sample preparation and MCC-IMS measurements were performed for each non-resistant strain and control at three different days (day 1, *n* = 5; day 2, *n* = 3; and day 3, *n* = 3). Ten replicates of each resistant strain were used.

### MCC-IMS analysis

**Fig. 1:**
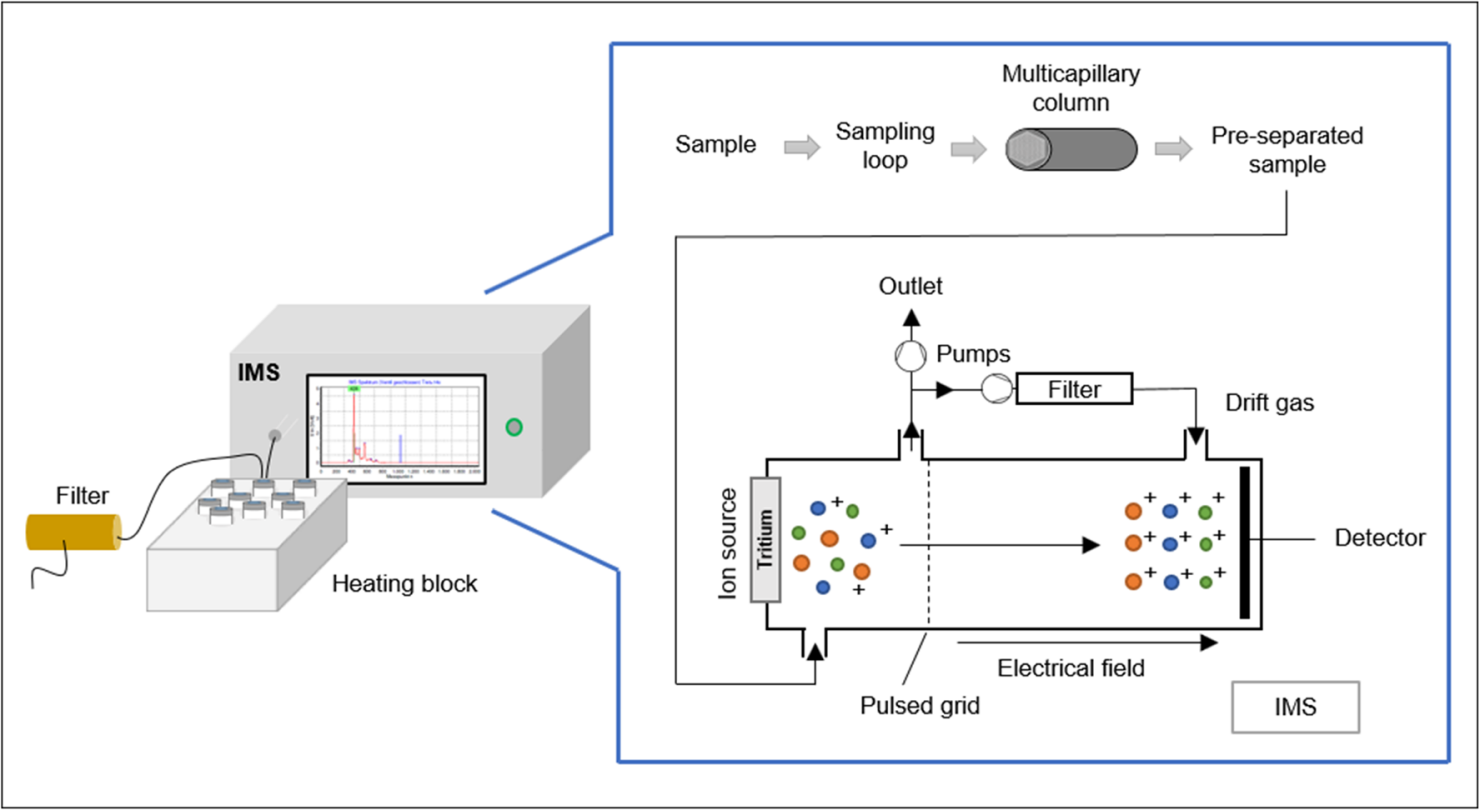
Schematic drawing of the setup for sampling and IMS measurement of bacterial cultures in headspace vials (modified from the manual, version 10.2017, of the used IMS device)

The VOCs emitted by bacteria into the headspace above cultures were analyzed with an ion mobility spectrometer coupled to a multi-capillary column (MCC-IMS) from STEP (Sensortechnik und Elektronik Pockau GmbH, Pockau, Germany). This device contains an internal gas circulation with a filter, which is regulated by a circulation pump to provide filtered ambient air as drift gas (400 ml/min) as well as analysis gas (20 ml/min). The whole experimental set up for this study is visualized in Fig. 1.

From the headspace of each sample vial, 10 ml were drawn via a 30-cm PTFE tube into a heated 0.7-ml sample loop (50 °C) by an internal pump with a flow rate of 200 ml/min. Therefore, the septum of the headspace vial was punctured with two cannulas. One cannula was used for VOC sampling and the other for pressure equalization in the headspace vial. The latter cannula is connected to an activated carbon filter to avoid interfering organic substances. After sampling, the gaseous analytes were pre-separated by the isothermally heated (40 °C) multi-capillary column (OV-5, 20 cm, Multichrom Ltd., Novosibirsk, Russia) and finally migrated into the IMS unit based on their retention times. There, they were initially ionized by a radioactive tritium source (99 MBq). Afterwards, the generated charged ions were accelerated by an electrical field (400 V/cm) towards the heated detector (60 °C) against the drift-gas flow. Due to collisions with drift gas molecules, ions were separated based on their mobility measured as drift time. The IMS can record positive or negative ions. For this study, the positive ion mode was applied. The basic working principle of IMS has been described in Stach and Baumbach (2002).

### MCC-IMS data analysis

**Fig. 2:**
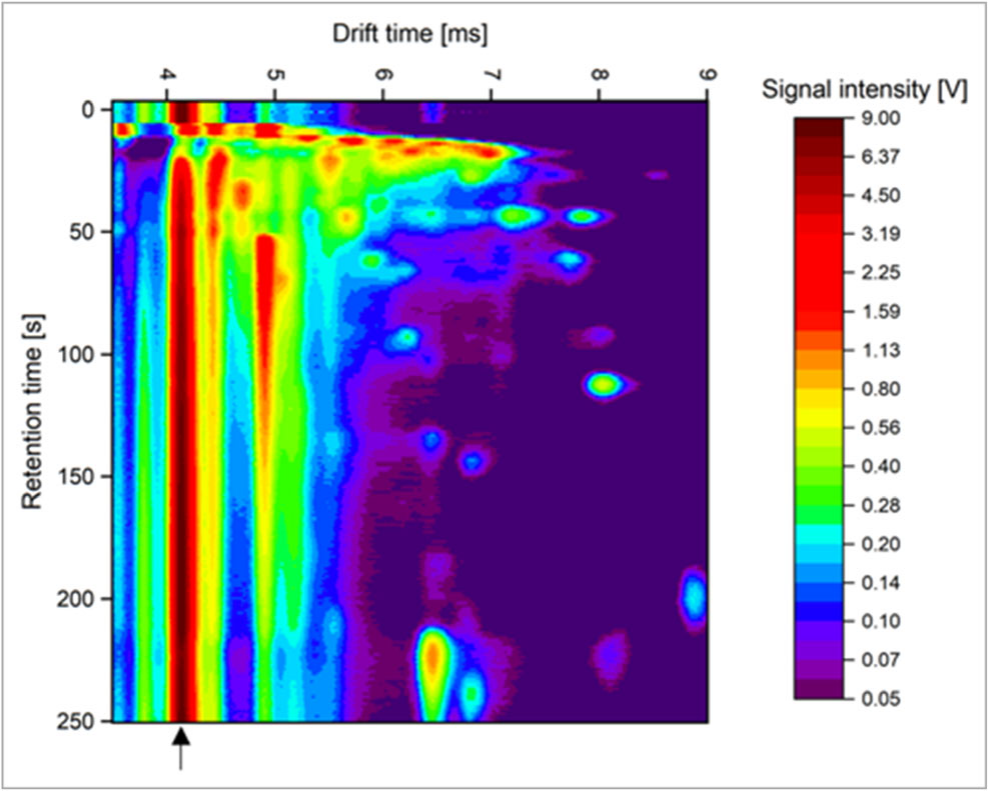
An exemplary MCC-IMS heatmap of positive charged components of volatile bacterial metabolites. Volatile components are depicted as peaks in signal intensity in volts (V) depending on the retention time in seconds (s) on the y-axis and drift time in milliseconds (ms) on the x-axis. The red-brown line (see arrow) at a drift time of 4.14 ms represents the reactant ion peak which originates from ions from the drift gas and does not correspond to analytes

The entire dataset consists of 86 measurements. During each measurement, 253 raw IMS spectra were recorded, i.e., one IMS spectrum at each second of MCC retention time. Within this time, all relevant headspace VOCs could be expected to be detected (Jünger et al. 2012; Kunze et al. 2013). All raw spectra together form a two-dimensional matrix where peaks are depicted as signal intensity in volts (V) depending on the retention time in seconds (s) on the *y*-axis and drift time in milliseconds (ms) on the *x*-axis as seen in Fig. 2. For data analysis, the pattern of peaks was considered. Thus, dimers and trimers have not been taken into account. All peaks together form the VOC pattern of the sample.

To differentiate between different bacterial strains based on their distinct VOC patterns, the raw MCC-IMS spectra of all measurements were processed with a proprietary cluster-analysis software. The analysis principle of this software is described in Purkhart et al. (2011), Purkhart (2010), and Becher et al. (2012). In brief, this software integrates data pre-processing steps (background adjustment, smoothing), peak detection based on local maxima calculation, and a hierarchical clustering algorithm to account for varying peak positions of the same VOC in different measurements (Purkhart et al. 2011; Purkhart 2010; Becher et al. 2012). This is based on the assumption that every cluster represents one VOC. The generated clusters can be used as a parameter for comparing sample groups as well as for statistical analysis. In this study, only peaks with a signal intensity higher than 0.05 V were registered.

### Statistical analysis

The resulting VOC clusters were analyzed using descriptive and multivariate statistical methods (IBM SPSS Statistics, Version 26.0, IBM Corp., Armonk, NY, USA; Stata Statistical Software: Release 11, StataCorp LP, College Station, TX, USA). Signal intensities in volt (V) of the VOC clusters were used as variables in this statistical analysis. The first step involved picking relevant VOC clusters of non-resistant and resistant bacterial strains. A VOC cluster was considered relevant if at least 80% of replicates had the peak and the median signal intensity was greater than control. To identify VOC clusters significantly differentiating each bacterial strain from control, Wilcoxon rank-sum test was applied. Values of *p* ≤ 0.05 were considered significant. With these significant VOC clusters, a canonical discriminant analysis (CDA) was performed. CDA was chosen because it is a widely used classification technique for IMS data and focuses on differences between groups (Szymańska et al. 2016). For visualization of differences, a three-dimensional (3D) scatterplot was created (Origin, Version 2018, OriginLab Corporation, MA, USA).

In order to investigate if each bacterial strain can be classified based on only some specific peaks, a hierarchical classification tree was constructed. This tree could be used to separate bacterial classes based on specific VOC clusters. The receiver-operating characteristic (ROC) curve analysis was applied to find classifiers (VOC clusters) for separation of bacterial classes for the classification tree. Variables used for the ROC analysis were the VOC clusters for which each bacterial strain showed a higher signal intensity compared to control medium (Wilcoxon rank-sum test with *p* ≤ 0.05) and at least 80% of their replicates had the peak. The performance of the classifier is determined by the area under curve (AUC). The higher the AUC, the better is the prediction of the classifier. The VOC clusters with highest AUC were selected as classifiers, or decision nodes in the tree. Additionally, different candidate threshold values of signal intensity [V] for a classifier could be obtained by ROC analysis. The threshold value with the highest sensitivity and specificity was used as cut point value for branching in the hierarchical tree.

## Results

The headspace of non-resistant bacterial strains and the control was investigated by applying MCC-IMS on three different days to ensure the reproducibility of the method. A number of 90 VOC clusters from resistant and non-resistant bacterial strains as well as controls had a signal in at least 80% of replicates and were considered further. Subsequently, VOC clusters with a significantly higher signal in bacteria samples compared to the control were selected assuming that these VOCs were produced by the bacteria. As a result, 63 VOC clusters were identified. Their retention and drift times are listed in Table 1 and their positions in the two-dimensional plot in supplemental Figure S1.

**Table 1:** List of 63 VOC clusters for which non-resistant and resistant bacterial strains show a significantly higher signal intensity compared to control medium (Wilcoxon rank-sum test with *p* ≤ 0.05) and at least 80% of their replicates have the peak. The VOC clusters are listed with their retention time (RT) in seconds (s) and drift time (td) in milliseconds (ms) as well as their occurrence (x) in the investigated seven different bacterial strains

			Non-resistant bacterial strains					Resistant bacterial strains	
VOC cluster	RT (s)	td (ms)	EC	MSSA	PA	KP	AB	MRSA	KP ESBL
c_1	2	5.28						x	
c_2	6	4.49	x			x			x
c_3	8	5.01	x			x		x	x
c_4	9	4.75			x				
c_5	11	5.37						x	x
c_6	12	5.75				x			x
c_7	13	6.18						x	
c_8	17	6.04	x	x				x	x
c_9	17	6.33	x			x	x		x
c_10	17	6.88			x		x		
c_11	18	7.02	x			x			x
c_12	20	5.62	x			x	x		x
c_13	21	4.55	x		x	x			x
c_14	27	5.20						x	
c_15	27	6.88					x		
c_16	32	6.30		x					
c_17	34	4.75		x	x			x	x
c_18	39	5.20						x	
c_19	39	6.01	x						x
c_20	42	6.44				x			x
c_21	43	6.29			x				
c_22	43	6.87				x			
c_23	43	7.31				x			
c_24	43	7.88				x			
c_25	44	5.73				x		x	
c_26	46	5.20		x					
c_27	49	4.47	x			x			x
c_28	53	7.69						x	
c_29	57	4.97						x	x
c_30	57	6.66		x				x	
c_31	60	6.78							x
c_32	60	7.14						x	x
c_33	62	5.95					x		
c_34	67	5.54	x						
c_35	75	4.96						x	
c_36	79	7.13							x
c_37	83	5.54	x			x			x
c_38	88	5.94							x
c_39	90	6.66		x					
c_40	91	7.13						x	
c_41	93	6.23					x		
c_42	96	5.56	x			x			x
c_43	98	7.14						x	
c_44	106	5.55	x						x
c_45	111	8.15					x		
c_46	115	4.98						x	
c_47	134	6.96						x	
c_48	135	5.64		x				x	
c_49	145	5.55							x
c_50	162	5.22		x				x	
c_51	191	5.23						x	
c_52	208	6.82	x						
c_53	212	5.60						x	x
c_54	223	5.57	x						x
c_55	223	8.15				x			
c_56	225	6.53			x	x			
c_57	229	5.54	x			x			x
c_58	236	5.53	x						x
c_59	238	4.98						x	x
c_60	239	5.23						x	
c_61	243	5.23						x	
c_62	243	5.54	x						x
c_63	245	4.97						x	x

The contribution of each single VOC cluster to the differentiation between non-resistant and resistant bacterial strains is shown in Table 1. It is evident that all seven bacterial strains can be distinguished based on these 63 VOC clusters. Most VOC clusters were found for resistant strains, ESBL producing *K. pneumoniae* (29) and methicillin-resistant *S. aureus* (26). Non-resistant *K. pneumoniae* (18) and *E. coli* (18) generated more VOCs compared to the other non-resistant strains methicillin-sensitive *S. aureus* (8), *A. baumannii* (7), and *P. aeruginosa* (6). Some VOC clusters, e.g., c_52 and c_55, could be assigned to only one strain but others like c_2, c_8, and c_10 to several strains, respectively. Interestingly, *E. coli* shared several VOC clusters with the two strains of *K. pneumoniae* that are not present in the headspace of control or the other bacterial strains (see supplemental Figure S2).

**Fig. 3:**
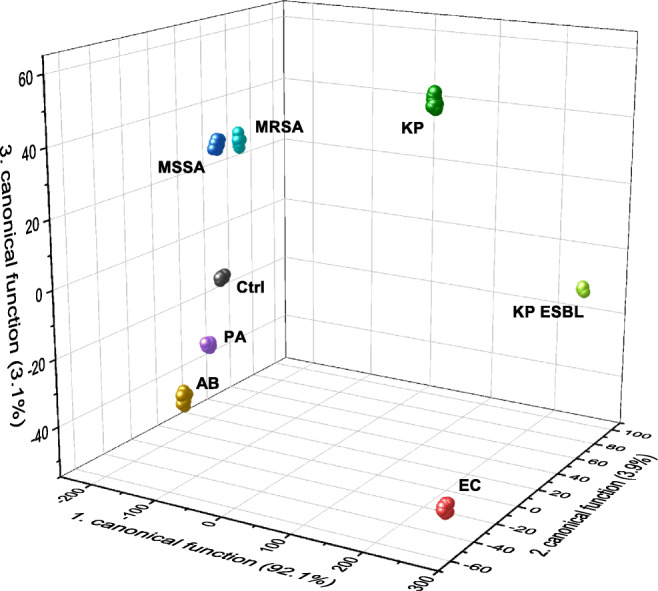
All 86 measurements from the different bacterial strains and the control are displayed as single data points in a three-dimensional space spanned by the first three canonical functions. These functions are linear combinations of the selected 63 VOC clusters and are explaining already 99.1% of the variance (first, second, and third canonical function: 92.1%, 3.9%, and 3.1%, respectively). The plot shows a clear discrimination between non-resistant and resistant bacterial strains. Ctrl Control, EC E. coli, MSSA methicillin-sensitive *S. aureus*, PA *P. aeruginosa*, KP *K. pneumoniae*, AB *A. baumannii*, MRSA methicillin-resistant *S. aureus*, KP ESBL extended-spectrum beta-lactamase producing *K. pneumoniae*

To determine the discrimination between the seven bacterial strains, a canonical discriminant analysis was performed. To visualize the results, a three-dimensional plot (Fig. 3) was created for the first three canonical functions using the selected 63 VOC clusters. In this plot, bacterial strains and controls are represented as colored data points (one data point for each replicate). The presented three canonical functions explain a variance of 99.1%. Figure 3 displays a clear differentiation between all seven bacterial strains including two resistant strains from the control medium. Within the bacterial strains, a separation with highest distances was found for *E. coli* as well as for both strains of *K. pneumoniae*. In contrast, the data points of *S. aureus* (MSSA) and resistant *S. aureus* (MRSA) are in closer proximity. Based on the classification model, all bacterial strains can be assigned to their group 100% correctly by using leave-one-out cross-validation (see supplemental Table S1).

**Fig. 4:**
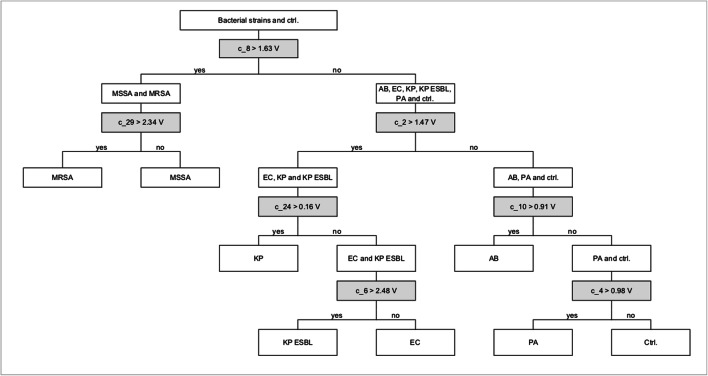
Hierarchical classification tree with seven VOC clusters. This tree allows for a classification of the investigated bacterial strains using specific VOC clusters. Grey boxes present the VOC clusters with cut point value in volt (V) serving as decision variable for splitting respective classes into sub-classes. Ctrl. Control, EC *E. coli*, MSSA methicillin-sensitive *S. aureus*, PA *P. aeruginosa*, KP *K. pneumoniae*, AB *A. baumannii*, MRSA methicillin-resistant *S. aureus*, KP ESBL extended-spectrum beta-lactamase producing *K. pneumoniae*

**Fig. 5:**
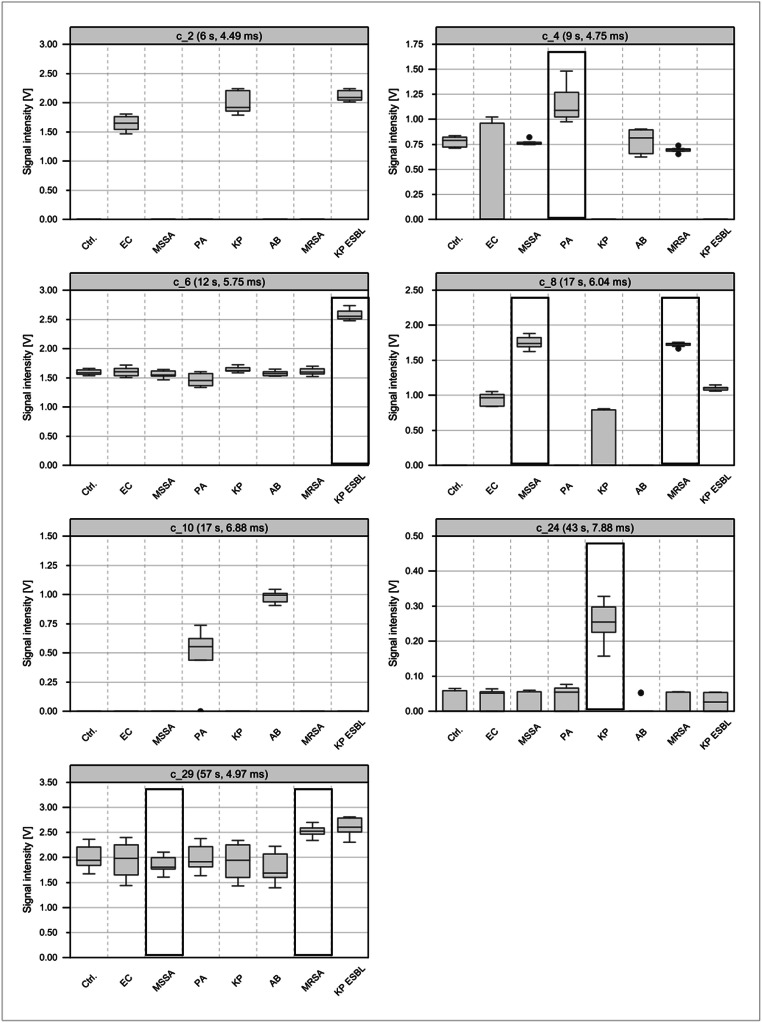
Boxplots of seven VOC clusters that allow a classification of different bacterial strains by using classification tree approach. EC *E. coli*, MSSA methicillin-sensitive *S. aureus*, PA *P. aeruginosa*, KP *K. pneumoniae*, AB *A. baumannii*, MRSA methicillin-resistant *S. aureus*, KP ESBL extended-spectrum beta-lactamase producing *K. pneumoniae*

Alternatively, to the canonical discriminant functions, a hierarchical classification tree was constructed. This classification tree can be used to separate the investigated bacterial classes based on specific VOC clusters, which were selected from the 63 VOC clusters presented in Table 1. By ROC curve analysis the separation of the different bacterial strains could be achieved with an AUC of 1.0. For several bacterial strains, more than one VOC cluster could be used for the dichotomization (see supplemental material). The resulting hierarchical tree with seven VOC clusters (Fig. 4) allows a classification of all bacterial strains. Using cluster c_8 with a cut point of 1.63 V, two classes, the staphylococci and the gram-negative bacteria, were differentiated. Depending on the cluster 2 with a cut point of 1.47 V, gram-negative bacteria could be separated into fermenting (*E*. *coli*, *K. pneumoniae*, and ESBL producing *K. pneumoniae*) and non-fermenting bacteria (*A. baumannii* and *P. aeruginosa*). Furthermore, the classification into subsequent classes could be performed by specific VOC clusters according to Fig. 4. In Fig. 5, seven VOC clusters are shown as boxplots that enable classification of certain bacterial strains by using ROC curve analyses.

## Discussion

This study shows that seven bacterial strains including resistant strains can be differentiated by applying MCC-IMS in the headspace of liquid cultures after 90-min incubation. The 63 VOC clusters listed in Table 1 were used as input variables for a canonical discriminant analysis. The discriminant analysis separated and classified the clusters correctly with a success rate of 100%. Moreover, using seven VOC clusters, a hierarchical classification tree was able to distinguish all investigated bacterial strains with an AUC of 1.0 by ROC curve analysis. Nevertheless, it has to be considered that the classification tree is prone to overfitting and might not be suitable for real and unknown data as only one strain per species was investigated. Therefore, the results need to be validated on patient derived samples.

Only few studies used IMS to explore the feasibility of fast bacteria identification. Drees et al. (2019) investigated VOCs emitted by *E. coli*, *S. aureus*, and *P. aeruginosa* in headspace of blood cultures using GC-IMS hourly for up to 8 h of incubation. Their results revealed that the best differentiation could be achieved after 6 h of incubation. Kunze et al. (2013) studied the VOCs related to the growth of *E. coli* and *P. aeruginosa* in lysogenic broth as a culture medium using MCC-IMS. The differences between them were observed in the logarithmic and stationary phase. Compared to these two studies, we achieved a differentiation as early as 90 min after inoculation. It can be assumed that a higher concentration of bacteria may lead to an earlier occurrence of VOCs and therefore to an earlier differentiation by headspace analysis with IMS. Unfortunately, Drees et al. (2019) and Kunze et al. (2013) did not specify the start concentration of the bacteria cultures, although it is apparent that both studies used an initial bacteria suspension with a much lower OD. Therefore, the differentiation in such an early stage in our study might be due to the high initial bacteria concentrations. In addition, the OD value of 0.1 used in our study results in a quite high bacteria concentration and might not reflect cell numbers in clinical settings.

With IMS positive and negative ions can be detected. We used the positive ion modes only. The need for negative ions may depend on the amount of VOCs present after different incubation times. For the differentiation of 15 different human pathogenic bacteria after 24 h of incubation, Jünger et al. (2012) required the negative ion mode in addition to the positive one. Our restriction to positive ion mode is in line with the study of Drees et al. (2019) where only one VOC in negative ion mode could be detected for *E. coli* within the first 8 h of incubation. This aspect could be confirmed by an earlier study (Steppert 2013) in which the negative ion mode rarely delivered information compared to the positive ion mode. Moreover, this study showed that the number of bacterial VOCs dropped drastically after 6 h of incubation (Steppert 2013). It may be assumed that after 24 h of incubation, there was a substrate depletion leading to lower concentration of VOCs.

This study focused on VOCs, which are produced by the bacteria. Therefore, only VOC clusters with a higher intensity in bacterial samples compared to the culture medium control were included. There might be even more relevant VOC clusters if clusters would have been considered that exhibit a lower intensity in bacterial samples than control samples indicating consumption of culture medium components.

Our data demonstrated that some VOCs are released not only by one but also by different bacterial strains. Our findings are in accordance with Boots et al. (2014) who revealed that one specific VOC may not be sufficient for distinguishing different bacterial species. Moreover, the released bacterial VOCs might also depend on available nutrients or growth medium (Küntzel et al. 2016; Ratiu et al. 2017). Thus, for a reliable differentiation, further studies need to be performed.

The obtained hierarchical classification tree supports the hypothesis that the differentiation by VOCs depends on the metabolic features of the different bacteria. Thus, we assume that the use of bacterial VOCs for species identification can be applied similar to the principle of the analytical profile index (API) system. The API system relies on classifying bacteria based on metabolic characteristics in an array of biochemical tests. For example, the differentiation between *Enterobacteriaceae* and non-*Enterobacteriaceae* within gram-negative rods depends on the presence or absence of oxidase and therefore on the fermentation ability of sugars. In our study, several distinct VOCs appeared that differ between fermenters and non-fermenters. However, the presented classification tree applies only for the bacterial species investigated in this study. In order to confirm the above-mentioned hypothesis, more members of different bacteria families will have to be investigated.

Several studies that have been published as conference abstracts only could also show that even resistant strains can be differentiated from non-resistant strains by IMS (Steppert et al. 2018; Becher et al. 2016). The discrimination of resistant bacteria by means of their VOCs was also confirmed by other studies with mass spectrometry (Boots et al. 2014; Rees et al. 2018). It remains unclear, if the difference of VOC patterns originates from metabolic shift induced by the resistance mechanism itself or from using two different strains for resistant and susceptible bacteria with different metabolic features. To clarify this, other approaches than VOC pattern recognition should be considered since the knowledge of the identity of the differentiating VOCs might help if these could be connected to certain metabolic processes. However, chemical identification with IMS is laborious and reference measurements are needed. Nonetheless, similar results in several studies imply that the VOC difference between MSSA and MRSA as well as *K. pneumoniae* versus extended-spectrum beta-lactamase producing *K. pneumoniae* may be based on an altered metabolism triggered by the resistance mechanism. In the other studies (Steppert et al. 2018; Becher et al. 2016; Steppert 2013), this was consistently found in clinical isolates. It can be assumed that different resistant mechanisms like cleaving of antibiotics may also produce different volatile metabolites compared to non-resistant strains (Tenover 2006). To answer this question conclusively, studies with strains before and after transfections of resistance genes should be undertaken. Therefore, the VOC pattern recognition approach might even be sufficient without the need of substance identification, although the latter still might give more background information.

In most VOC analysis studies, offline methods like solid phase microextraction (SPME) and desorption tubes are used to sample VOCs (Wilde et al. 2019; Pereira et al. 2014). With regard to on-site screening, those methods are quite elaborate due to a long enrichment period of about 30 min and require an additional thermal desorption unit to release VOCs for analysis. Therefore, we chose direct analysis by drawing the VOCs into the system by an internal pump. Altogether, sampling and analysis took only 4 min. In addition, this method does not require expert knowledge and is convenient due to easy handling and a low waiting time required to receive the results. Compared to other IMS devices, the utilized STEP-IMS device does not need synthetic gases. This reduces consumables and enables on-site analyses.

Compared to Drees et al. (2019) and Jünger et al. (2012), we did not perform mass spectrometry in addition to IMS to identify the VOCs chemically, although this might be helpful to explain the origin of the VOCs. However, at this stage, the aim of the study was to show the identification of bacteria based on VOC patterns. There are successful attempts to train animals like dogs and rats to smell infections of resistant bacteria and tuberculosis, respectively (Koivusalo et al. 2017; Fiebig et al. 2020). Identification of VOCs by animals relies on the recognition of VOC patterns or a number of specific VOCs of this pattern that are responsible for the scents. Hence, it should be possible to show the identification of bacteria based on certain compositions of VOC clusters in the form of patterns without knowing the substance identity of each single VOC.

To support a reliable analysis with MCC-IMS, we measured the same bacterial strain several times on three different days in order to obtain the most distinctive VOC clusters and to eliminate random influences. Nevertheless, only reference strains in small sample sizes were investigated which constitutes a limitation of this study. Moreover, the clinical relevance of these reference strains is limited. Further investigations on clinical isolates of different bacterial species on a greater scale should be conducted to confirm the ability of MCC-IMS method to identify specific species based on VOC patterns.

In conclusion, this study demonstrates that MCC-IMS was able to differentiate reference strains of seven different bacterial species including resistant species based on VOC patterns after 90 min of incubation by applying canonical discriminant analysis. Sampling and MCC-IMS- analysis took only 4 min. Moreover, the study shows that discriminative VOCs can be assigned to specific species based on their metabolic characteristics using a classification tree, even without knowing the substance identity.

MCC-IMS may become a reliable, cost-effective, and rapid on-site screening tool for infectious pathogens. In future studies, not only different bacterial species but also viruses as well as breath should be investigated under the influence of different methodological factors.

## Supplementary information

ESM 1 (PDF 3090 kb)
