# Supplementary material for: Rapid in vitro differentiation of bacteria by ion mobility spectrometry
Source: Appl Microbiol Biotechnol. 2021 May 11;105(10):4297–307. doi: 10.1007/s00253-021-11315-w (PMC8140968; doi:10.1007/s00253-021-11315-w)
Supplement: Supplementary file 1 — (PDF 3090 kb) [file 253_2021_11315_MOESM1_ESM.pdf]

Supplementary Material

Rapid in-vitro differentiation of bacteria by ion mobility spectrometry

Journal: Applied Microbiology and Biotechnology

Isabel Steppert [1,2], Jessy Schönfelder [1, 3,*], Carolyn Schultz [1], Dirk Kuhlmeier [1,3]

1 Fraunhofer Institute for Cell Therapy and Immunology IZI, MicroDiagnostics, Leipzig, Germany
2 University of Potsdam, Institute for Biochemistry and Biology, Potsdam, Germany
3 Fraunhofer Institute for Cell Therapy and Immunology IZI, Project Hub Microelectronic and Optical Systems for Biomedicine MEOS, Erfurt, Germany

*corresponding author (jessy.schoenfelder@izi.fraunhofer.de)

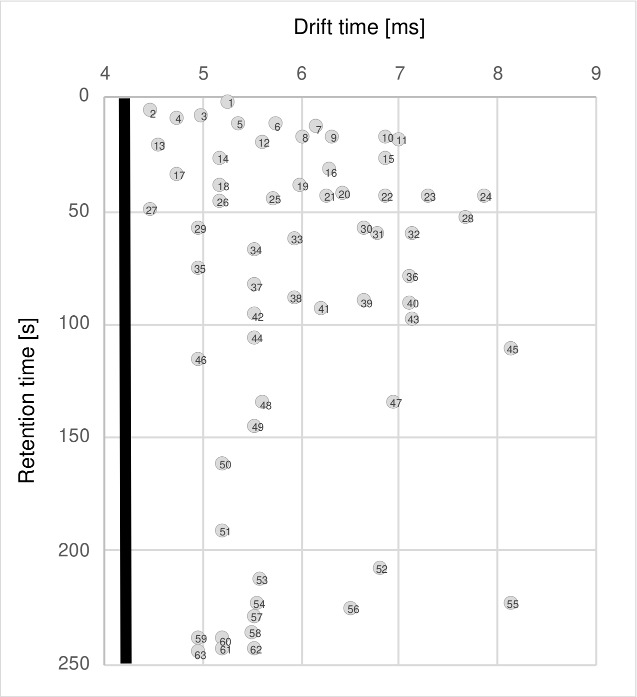

Figure S1 Two-dimensional IMS plot with the position of 63 selected VOC clusters which were analyzed for this study.

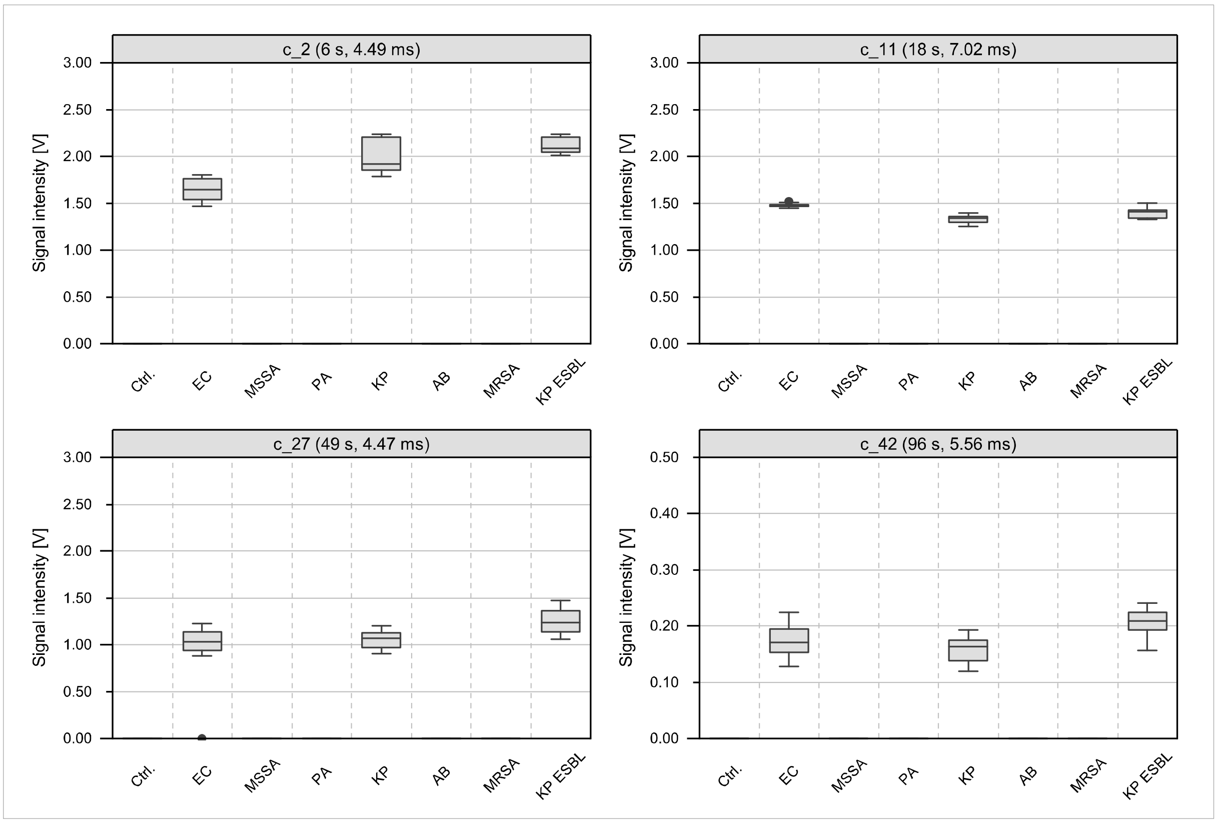

Figure S2 Boxplots of four exemplary VOC clusters which are shared by *E. coli*, *K. pneumoniae* and ESBL producing *K. pneumoniae,* whereas the other strains and control show no signal. Ctrl = Control; EC = *E. coli*; KP = *K. pneumoniae* (sensitive); KP ESBL = ESBL producing *K. pneumoniae* (resistant); AB = *A. baumannii*; PA = *P. aeruginosa*; MSSA = Methicillin sensitive *S. aureus*; MRSA = Methicillin resistant *S. aureus.* n =10-11.

Table S1 Classification results of canonical discriminant analysis with 63 VOC clusters. Ctrl = Control; EC = *E. coli*; MSSA = Methicillin sensitive *S. aureus*; PA = *P. aeruginosa*; KP = *K. pneumoniae* (sensitive); AB = *A. baumannii*; MRSA = Methicillin resistant *S. aureus*; KP ESBL = Extended-spectrum beta-lactamase producing *K. pneumoniae* (resistant).

Classification Results

			Predicted Group Membership								Total
		Group	Ctrl	EC	MSSA	PA	KP	AB	MRSA	KP ESBL	
Original	Count	Ctrl	11	0	0	0	0	0	0	0	11
		EC	0	11	0	0	0	0	0	0	11
		MSSA	0	0	11	0	0	0	0	0	11
		PA	0	0	0	11	0	0	0	0	11
		KP	0	0	0	0	11	0	0	0	11
		AB	0	0	0	0	0	11	0	0	11
		MRSA	0	0	0	0	0	0	10	0	10
		KP ESBL	0	0	0	0	0	0	0	10	10
	%	Ctrl	100	0	0	0	0	0	0	0	100
		EC	0	100	0	0	0	0	0	0	100
		MSSA	0	0	100	0	0	0	0	0	100
		PA	0	0	0	100	0	0	0	0	100
		KP	0	0	0	0	100	0	0	0	100
		AB	0	0	0	0	0	100	0	0	100
		MRSA	0	0	0	0	0	0	100	0	100
		KP ESBL	0	0	0	0	0	0	0	100	100
Cross-validated	Count	Ctrl	11	0	0	0	0	0	0	0	11
		EC	0	11	0	0	0	0	0	0	11
		MSSA	0	0	11	0	0	0	0	0	11
		PA	0	0	0	11	0	0	0	0	11
		KP	0	0	0	0	11	0	0	0	11
		AB	0	0	0	0	0	11	0	0	11
		MRSA	0	0	0	0	0	0	10	0	10
		KP ESBL	0	0	0	0	0	0	0	10	10
	%	Ctrl	100	0	0	0	0	0	0	0	100
		EC	0	100	0	0	0	0	0	0	100
		MSSA	0	0	100	0	0	0	0	0	100
		PA	0	0	0	100	0	0	0	0	100
		KP	0	0	0	0	100	0	0	0	100
		AB	0	0	0	0	0	100	0	0	100
		MRSA	0	0	0	0	0	0	100	0	100
		KP ESBL	0	0	0	0	0	0	0	100	100

100.0% of original grouped cases correctly classified.

100.0% of cross-validated grouped cases correctly classified.

Results of ROC curve analysis

1) Classifier for the differentiation between gram-positive and gram-negative bacteria

Classifier for Staphylococci (MSSA and MRSA)

5 variables: c_8 c_17 c_30 c_48 c_50

	Obs	ROC Area	Std. Err.	Asymptotic Normal [95% Conf.	Interval]
c_8	86	1.0000	0.0000	1.00000	1.00000
c_17	86	0.5758	0.0603	0.45760	0.69404
c_30	86	0.9586	0.0322	0.89556	1.00000
c_48	86	0.9513	0.0224	0.90738	0.99519
c_50	86	0.9319	0.0275	0.87802	0.98571

c_8 as classifier with AUC of 1.0

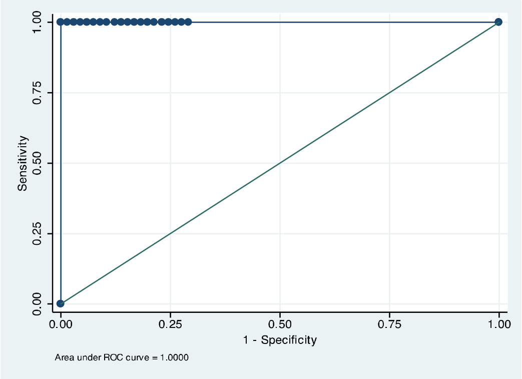

c_8 as classifier

Cutpoint	Sensitivity	Specificity	Correctly Classified	LR+	LR-
(>= 0)	100.00%	0.00%	24.42%	1.0000	
(>= .793)	100.00%	0.00%	24.42%	1.0000	
(>= .795)	100.00%	63.08%	72.09%	2.7083	0.0000
(>= .807)	100.00%	64.62%	73.26%	2.8261	0.0000
(>= .841)	100.00%	66.15%	74.42%	2.9545	0.0000
(>= .845)	100.00%	67.69%	75.58%	3.0952	0.0000
(>= .846)	100.00%	69.23%	76.74%	3.2500	0.0000
(>= .849)	100.00%	70.77%	77.91%	3.4211	0.0000
(>= .86)	100.00%	72.31%	79.07%	3.6111	0.0000
(>= .965)	100.00%	75.38%	81.40%	4.0625	0.0000
(>= .985)	100.00%	76.92%	82.56%	4.3333	0.0000
(>= 1.003)	100.00%	78.46%	83.72%	4.6429	0.0000
(>= 1.01)	100.00%	80.00%	84.88%	5.0000	0.0000
(>= 1.018)	100.00%	81.54%	86.05%	5.4167	0.0000
(>= 1.052)	100.00%	83.08%	87.21%	5.9091	0.0000
(>= 1.061)	100.00%	84.62%	88.37%	6.5000	0.0000
(>= 1.071)	100.00%	86.15%	89.53%	7.2222	0.0000
(>= 1.076)	100.00%	87.69%	90.70%	8.1250	0.0000
(>= 1.079)	100.00%	89.23%	91.86%	9.2857	0.0000
(>= 1.105)	100.00%	90.77%	93.02%	10.8333	0.0000
(>= 1.109)	100.00%	92.31%	94.19%	13.0000	0.0000
(>= 1.111)	100.00%	93.85%	95.35%	16.2500	0.0000
(>= 1.113)	100.00%	95.38%	96.51%	21.6667	0.0000
(>= 1.127)	100.00%	96.92%	97.67%	32.5000	0.0000
(>= 1.147)	100.00%	98.46%	98.84%	65.0001	0.0000
(>= 1.627)	100.00%	100.00%	100.00%		0.0000
(>= 1.666)	95.24%	100.00%	98.84%		0.0476
(>= 1.689)	90.48%	100.00%	97.67%		0.0952
(>= 1.693)	80.95%	100.00%	95.35%		0.1905
(>= 1.696)	76.19%	100.00%	94.19%		0.2381
(>= 1.713)	71.43%	100.00%	93.02%		0.2857
(>= 1.715)	66.67%	100.00%	91.86%		0.3333
(>= 1.723)	61.90%	100.00%	90.70%		0.3810
(>= 1.734)	52.38%	100.00%	88.37%		0.4762
(>= 1.738)	42.86%	100.00%	86.05%		0.5714
(>= 1.739)	33.33%	100.00%	83.72%		0.6667
(>= 1.741)	28.57%	100.00%	82.56%		0.7143
(>= 1.742)	23.81%	100.00%	81.40%		0.7619
(>= 1.752)	19.05%	100.00%	80.23%		0.8095
(>= 1.756)	9.52%	100.00%	77.91%		0.9048
(>= 1.769)	4.76%	100.00%	76.74%		0.9524
(>= 1.824)	0.00%	100.00%	75.58%		1.0000
(>= 1.864)	100.00%	73.85%	80.23%	3.8235	0.0000
(>= 1.882)	.%	.%	.%		
(> 1.882)	.%	.%	.%		

Obs	ROC Area	Std. Err.	—Asymptotic Normal— [95% Conf.	Interval]
86	1.0000	0.0000	1.00000	1.00000

2) Classifier for the differentiation between MSSA and MRSA

Classifier for MRSA

25 variables: c_1 c_3 c_5 c_7 c_14 c_17 c_18 c_25 c_28 c_29 c_30 c_32 c_35 c_40 c_43 c_46 c_47 c_48 c_50 c_51 c_53 c_59 c_60 c_61 c_63

	Obs	ROC Area	Std. Err.	—Asymptotic Normal— [95% Conf.	Interval]
c_1	21	0.7182	0.1164	0.49000	0.94636
c_3	21	0.8955	0.0704	0.75741	1.00000
c_5	21	1.0000	0.0000	1.00000	1.00000
c_7	21	0.9545	0.0479	0.86064	1.00000
c_14	21	0.6091	0.1299	0.35441	0.86377
c_17	21	0.7727	0.1065	0.56402	0.98143
c_18	21	0.6045	0.1321	0.34569	0.86341
c_25	21	0.8636	0.0794	0.70801	1.00000
c_28	21	0.8727	0.0779	0.72003	1.00000
c_29	21	1.0000	0.0000	1.00000	1.00000
c_30	21	0.6318	0.1279	0.38111	0.88252
c_32	21	0.9818	0.0219	0.93891	1.00000
c_35	21	1.0000	0.0000	1.00000	1.00000
c_40	21	0.8000	0.1008	0.60235	0.99765
c_43	21	0.8136	0.0968	0.62396	1.00000
c_46	21	0.8545	0.0982	0.66200	1.00000
c_47	21	0.4864	0.1343	0.22308	0.74965
c_48	21	0.3409	0.1319	0.08244	0.59938
c_50	21	0.8045	0.0990	0.61058	0.99852
c_51	21	0.8364	0.0896	0.66066	1.00000
c_53	21	0.8636	0.0838	0.69936	1.00000
c_59	21	0.8455	0.1042	0.64117	1.00000
c_60	21	0.8455	0.0886	0.67184	1.00000
c_61	21	0.8636	0.0828	0.70142	1.00000
c_63	21	0.9909	0.0129	0.96571	1.00000

Classifiers with AUC of 1.0: c_5 c_29 c_35

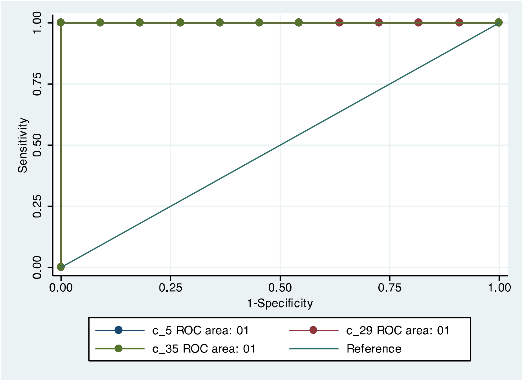

c_29 as classifier

Cutpoint	Sensitivity	Specificity	Correctly Classified	LR+	LR-
(>= 1.607)	100.00%	0.00%	47.62%	1.0000	
(>= 1.746)	100.00%	9.09%	52.38%	1.1000	0.0000
(>= 1.766)	100.00%	18.18%	57.14%	1.2222	0.0000
(>= 1.783)	100.00%	27.27%	61.90%	1.3750	0.0000
(>= 1.799)	100.00%	36.36%	66.67%	1.5714	0.0000
(>= 1.805)	100.00%	45.45%	71.43%	1.8333	0.0000
(>= 1.902)	100.00%	54.55%	76.19%	2.2000	0.0000
(>= 1.968)	100.00%	63.64%	80.95%	2.7500	0.0000
(>= 1.994)	100.00%	72.73%	85.71%	3.6667	0.0000
(>= 2.094)	100.00%	81.82%	90.48%	5.5000	0.0000
(>= 2.106)	100.00%	90.91%	95.24%	11.0000	0.0000
(>= 2.343)	100.00%	100.00%	100.00%		0.0000
(>= 2.456)	90.00%	100.00%	95.24%		0.1000
(>= 2.465)	80.00%	100.00%	90.48%		0.2000
(>= 2.472)	70.00%	100.00%	85.71%		0.3000
(>= 2.492)	60.00%	100.00%	80.95%		0.4000
(>= 2.56)	40.00%	100.00%	71.43%		0.6000
(>= 2.575)	30.00%	100.00%	66.67%		0.7000
(>= 2.592)	20.00%	100.00%	61.90%		0.8000
(>= 2.668)	10.00%	100.00%	57.14%		0.9000
(>= 2.701)	0.00%	100.00%	52.38%		1.0000
(> 2.701)	100.00%	0.00%	47.62%	1.0000	

Obs	ROC Area	Std. Err.	—Asymptotic Normal— [95% Conf.	Interval]
21	1.0000	0.0000	1.00000	1.00000

3) Classifier for the differentiation between gram-negative bacteria incl. control

Classifier for *Enterobacteriaceae* (*E. coli*, *K. pneumoniae* and ESBL producing *K. pneumoniae*)

10 variables: c_2 c_3 c_9 c_11 c_12 c_13 c_27 c_37 c_42 c_57

	Obs	ROC Area	Std. Err.	—Asymptotic Normal— [95% Conf.	Interval]
c_2	65	1.0000	0.0000	1.00000	1.00000
c_3	65	0.9602	0.0237	0.91383	1.00000
c_9	65	0.8082	0.0538	0.70275	0.91372
c_11	65	1.0000	0.0000	1.00000	1.00000
c_12	65	0.9991	0.0013	0.99643	1.00000
c_13	65	0.9673	0.0185	0.93101	1.00000
c_27	65	0.9844	0.0156	0.95375	1.00000
c_37	65	0.9375	0.0297	0.87929	0.99571
c_42	65	1.0000	0.0000	1.00000	1.00000
c_57	65	0.7836	0.0581	0.66974	0.89749

Classifiers with AUC of 1.0: c_2 c_11 c_42

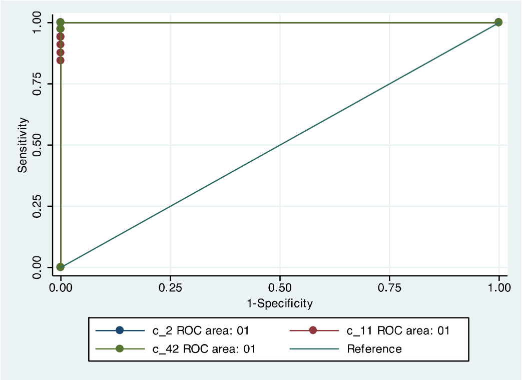

c_2 as classifier

Cutpoint	Sensitivity	Specificity	Correctly Classified	LR+	LR-
(>= 0)	100.00%	0.00%	49.23%	1.0000	
(>= 1.466)	100.00%	100.00%	100.00%		0.0000
(>= 1.478)	96.88%	100.00%	98.46%		0.0312
(>= 1.539)	93.75%	100.00%	96.92%		0.0625
(>= 1.564)	87.50%	100.00%	93.85%		0.1250
(>= 1.617)	84.38%	100.00%	92.31%		0.1562
(>= 1.645)	81.25%	100.00%	90.77%		0.1875
(>= 1.658)	78.12%	100.00%	89.23%		0.2188
(>= 1.71)	75.00%	100.00%	87.69%		0.2500
(>= 1.764)	68.75%	100.00%	84.62%		0.3125
(>= 1.768)	65.62%	100.00%	83.08%		0.3438
(>= 1.785)	62.50%	100.00%	81.54%		0.3750
(>= 1.807)	59.38%	100.00%	80.00%		0.4062
(>= 1.808)	56.25%	100.00%	78.46%		0.4375
(>= 1.855)	50.00%	100.00%	75.38%		0.5000
(>= 1.861)	46.88%	100.00%	73.85%		0.5312
(>= 1.887)	43.75%	100.00%	72.31%		0.5625
(>= 1.917)	40.62%	100.00%	70.77%		0.5938
(>= 2.01)	37.50%	100.00%	69.23%		0.6250
(>= 2.038)	31.25%	100.00%	66.15%		0.6875
(>= 2.041)	28.12%	100.00%	64.62%		0.7188
(>= 2.054)	25.00%	100.00%	63.08%		0.7500
(>= 2.081)	21.88%	100.00%	61.54%		0.7812
(>= 2.101)	18.75%	100.00%	60.00%		0.8125
(>= 2.116)	9.38%	100.00%	55.38%		0.9062
(>= 2.125)	6.25%	100.00%	53.85%		0.9375
(>= 2.191)	3.12%	100.00%	52.31%		0.9688
(>= 2.209)	0.00%	100.00%	50.77%		1.0000
(>= 2.215)	100.00%	0.00%	49.23%	1.0000	
(>= 2.223)	.%	.%	.%		
(>= 2.239)	.%	.%	.%		
(>= 2.242)	.%	.%	.%		
(> 2.242)	.%	.%	.%		

Obs	ROC Area	Std. Err.	—Asymptotic Normal— [95% Conf.	Interval]
65	1.0000	0.0000	1.00000	1.00000

4) Classifier for the differentiation between bacterial strains of *Enterobacteriaceae* (*E. coli*, *K. pneumoniae* and ESBL producing *K. pneumoniae*)

<u>Classifier for *K. pneumoniae*</u>

17 variables: c_3 c_6 c_9 c_11 c_12 c_13 c_20 c_22 c_23 c_24 c_25 c_27 c_37 c_42 c_55 c_56 c_57

	Obs	ROC Area	Std. Err.	—Asymptotic Normal— [95% Conf.	Interval]
c_3	32	0.6234	0.1018	0.42380	0.82296
c_6	32	0.3680	0.1012	0.16962	0.56631
c_9	32	0.7597	0.1050	0.55387	0.96561
c_11	32	0.1104	0.0584	0.00000	0.22478
c_12	32	0.0087	0.0104	0.00000	0.02909
c_13	32	0.5801	0.1113	0.36186	0.79831
c_20	32	0.9784	0.0215	0.93629	1.00000
c_22	32	0.8831	0.0789	0.72839	1.00000
c_23	32	1.0000	0.0000	1.00000	1.00000
c_24	32	1.0000	0.0000	1.00000	1.00000
c_25	32	1.0000	0.0000	1.00000	1.00000
c_27	32	0.3506	0.0982	0.15812	0.54318
c_37	32	0.2922	0.0953	0.10542	0.47900
c_42	32	0.2359	0.0844	0.07055	0.40131
c_55	32	0.9545	0.0455	0.86546	1.00000
c_56	32	1.0000	0.0000	1.00000	1.00000
c_57	32	0.2749	0.0942	0.09032	0.45947

Classifiers with AUC of 1.0: c_23 c_24 c_25 c_56

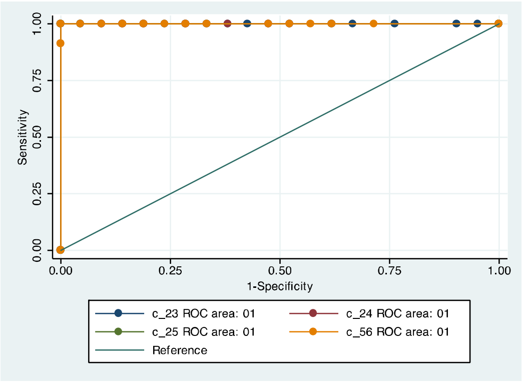

c_24 as classifier

Cutpoint	Sensitivity	Specificity	Correctly Classified	LR+	LR-
(>= 0)	100.00%	0.00%	34.38%	1.0000	
(>= .051)	100.00%	0.00%	34.38%	1.0000	
(>= .052)	100.00%	47.62%	65.62%	1.9091	0.0000
(>= .053)	100.00%	52.38%	68.75%	2.1000	0.0000
(>= .054)	100.00%	71.43%	81.25%	3.5000	0.0000
(>= .055)	100.00%	80.95%	87.50%	5.2500	0.0000
(>= .056)	100.00%	85.71%	90.63%	7.0000	0.0000
(>= .06)	100.00%	90.48%	93.75%	10.5000	0.0000
(>= .064)	100.00%	95.24%	96.88%	21.0000	0.0000
(>= .157)	100.00%	100.00%	100.00%		0.0000
(>= .181)	81.82%	100.00%	93.75%		0.1818
(>= .225)	72.73%	100.00%	90.62%		0.2727
(>= .23)	63.64%	100.00%	87.50%		0.3636
(>= .244)	54.55%	100.00%	84.38%		0.4545
(>= .255)	45.45%	100.00%	81.25%		0.5455
(>= .274)	27.27%	100.00%	75.00%		0.7273
(>= .278)	18.18%	100.00%	71.87%		0.8182
(>= .298)	9.09%	100.00%	68.75%		0.9091
(>= .303)	0.00%	100.00%	65.62%		1.0000
(>= .328)	100.00%	61.90%	75.00%	2.6250	0.0000
(> .328)	.%	.%	.%		

Obs	ROC Area	Std. Err.	—Asymptotic Normal— [95% Conf.	Interval]
32	1.0000	0.0000	1.00000	1.00000

5) Classifier for *E. coli* versus ESBL producing *K. pneumoniae*

<u>Classifier for ESBL producing *K. pneumoniae*</u>

27 variables: c_3 c_5 c_6 c_9 c_11 c_12 c_13 c_17 c_19 c_20 c_27 c_29 c_31 c_32 c_36 c_37 c_38 c_42 c_44 c_49 c_53 c_54 c_57 c_58 c_59 c_62 c_63

	Obs	ROC Area	Std. Err.	—Asymptotic Normal— [95% Conf.	Interval]
c_3	21	0.9545	0.0420	0.87229	1.00000
c_5	21	1.0000	0.0000	1.00000	1.00000
c_6	21	1.0000	0.0000	1.00000	1.00000
c_9	21	1.0000	0.0000	1.00000	1.00000
c_11	21	0.0818	0.0827	0.00000	0.24395
c_12	21	0.5091	0.1398	0.23506	0.78312
c_13	21	0.7727	0.1179	0.54174	1.00000
c_17	21	0.7455	0.1223	0.50578	0.98513
c_19	21	0.7227	0.1153	0.49667	0.94879
c_20	21	0.6773	0.1208	0.44044	0.91411
c_27	21	0.9000	0.0661	0.77045	1.00000
c_29	21	0.9818	0.0219	0.93891	1.00000
c_31	21	0.9318	0.0686	0.79736	1.00000
c_32	21	0.9045	0.0665	0.77430	1.00000
c_36	21	0.8818	0.0797	0.72556	1.00000
c_37	21	0.6864	0.1288	0.43383	0.93890
c_38	21	0.4091	0.1449	0.12506	0.69312
c_42	21	0.8045	0.1018	0.60508	1.00000
c_44	21	0.6682	0.1320	0.40953	0.92683
c_49	21	0.8727	0.0802	0.71554	1.00000
c_53	21	0.8545	0.0881	0.68192	1.00000
c_54	21	0.8273	0.0950	0.64104	1.00000
c_57	21	0.6409	0.1369	0.37266	0.90916
c_58	21	0.7182	0.1208	0.48148	0.95488
c_59	21	0.7818	0.1176	0.55125	1.00000
c_62	21	0.7091	0.1268	0.46053	0.95765
c_63	21	0.8909	0.0812	0.73172	1.00000

Classifiers with AUC of 1.0: c_5 c_6 c_9

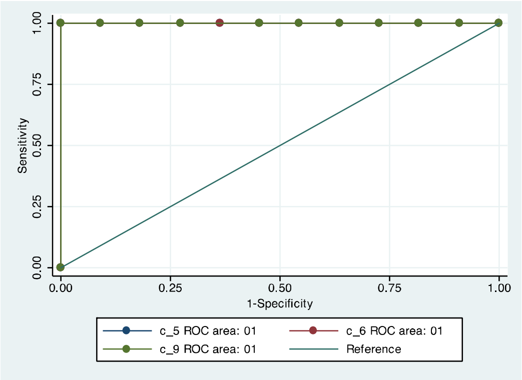

c_6 as classifier

Cutpoint	Sensitivity	Specificity	Correctly Classified	LR+	LR-
(>= 1.503)	100.00%	0.00%	47.62%	1.0000	
(>= 1.509)	100.00%	9.09%	52.38%	1.1000	0.0000
(>= 1.534)	100.00%	18.18%	57.14%	1.2222	0.0000
(>= 1.564)	100.00%	27.27%	61.90%	1.3750	0.0000
(>= 1.569)	100.00%	36.36%	66.67%	1.5714	0.0000
(>= 1.601)	100.00%	45.45%	71.43%	1.8333	0.0000
(>= 1.605)	100.00%	54.55%	76.19%	2.2000	0.0000
(>= 1.631)	100.00%	63.64%	80.95%	2.7500	0.0000
(>= 1.658)	100.00%	72.73%	85.71%	3.6667	0.0000
(>= 1.672)	100.00%	81.82%	90.48%	5.5000	0.0000
(>= 1.717)	100.00%	90.91%	95.24%	11.0000	0.0000
(>= 2.479)	100.00%	100.00%	100.00%		0.0000
(>= 2.504)	90.00%	100.00%	95.24%		0.1000
(>= 2.511)	80.00%	100.00%	90.48%		0.2000
(>= 2.528)	70.00%	100.00%	85.71%		0.3000
(>= 2.531)	60.00%	100.00%	80.95%		0.4000
(>= 2.572)	40.00%	100.00%	71.43%		0.6000
(>= 2.583)	30.00%	100.00%	66.67%		0.7000
(>= 2.639)	20.00%	100.00%	61.90%		0.8000
(>= 2.734)	10.00%	100.00%	57.14%		0.9000
(>= 2.735)	0.00%	100.00%	52.38%		1.0000
(> 2.735)	100.00%	0.00%	47.62%	1.0000	

Obs	ROC Area	Std. Err.	—Asymptotic Normal— [95% Conf. Interval]	
21	1.0000	0.0000	1.00000	1.00000

6) Classifier for *non-Enterobacteriaceae* (*A. baumannii*, *P. aeruginosa*)

Classifier for *A. baumannii*

7 variables: c_9 c_10 c_12 c_15 c_33 c_41 c_45

	Obs	ROC Area	Std. Err.	—Asymptotic Normal— [95% Conf.	Interval]
c_9	33	0.8967	0.0608	0.77749	1.00000
c_10	33	1.0000	0.0000	1.00000	1.00000
c_12	33	1.0000	0.0000	1.00000	1.00000
c_15	33	0.8616	0.0680	0.72821	0.99493
c_33	33	0.7500	0.0865	0.58056	0.91944
c_41	33	0.7004	0.0953	0.51365	0.88718
c_45	33	0.8430	0.0698	0.70610	0.97985

Classifiers with AUC of 1.0: c_10 c_12

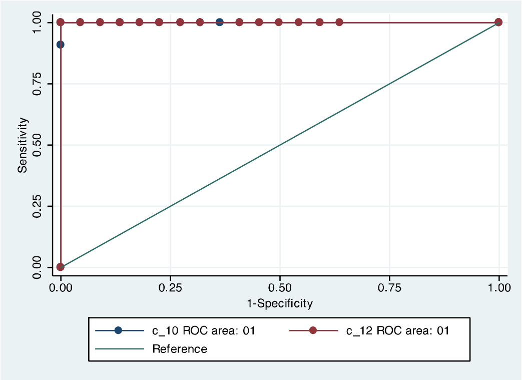

c_10 as classifier

Cutpoint	Sensitivity	Specificity	Correctly Classified	LR+	LR-
(>= 0)	100.00%	0.00%	33.33%	1.0000	
(>= .437)	100.00%	0.00%	33.33%	1.0000	
(>= .505)	100.00%	59.09%	72.73%	2.4444	0.0000
(>= .547)	100.00%	63.64%	75.76%	2.7500	0.0000
(>= .552)	100.00%	68.18%	78.79%	3.1429	0.0000
(>= .6)	100.00%	77.27%	84.85%	4.4000	0.0000
(>= .603)	100.00%	81.82%	87.88%	5.5000	0.0000
(>= .623)	100.00%	86.36%	90.91%	7.3333	0.0000
(>= .663)	100.00%	90.91%	93.94%	11.0000	0.0000
(>= .736)	100.00%	95.45%	96.97%	22.0000	0.0000
(>= .905)	100.00%	100.00%	100.00%		0.0000
(>= .921)	90.91%	100.00%	96.97%		0.0909
(>= .937)	81.82%	100.00%	93.94%		0.1818
(>= .945)	72.73%	100.00%	90.91%		0.2727
(>= .974)	63.64%	100.00%	87.88%		0.3636
(>= .995)	54.55%	100.00%	84.85%		0.4545
(>= .998)	36.36%	100.00%	78.79%		0.6364
(>= .999)	27.27%	100.00%	75.76%		0.7273
(>= 1.011)	18.18%	100.00%	72.73%		0.8182
(>= 1.02)	9.09%	100.00%	69.70%		0.9091
(>= 1.045)	0.00%	100.00%	66.67%		1.0000
(> 1.045)	100.00%	72.73%	81.82%	3.6667	0.0000

Obs	ROC Area	Std. Err.	—Asymptotic Normal— [95% Conf.	Interval]
33	1.0000	0.0000	1.00000	1.00000

7) Classifier for P. aeruginosa vs. Control

Classifier for *P. aeruginosa*

6 variables: c_4 c_10 c_13 c_17 c_21 c_56

	Obs	ROC Area	Std. Err.	—Asymptotic Normal— [95% Conf.	Interval]
c_4	22	1.0000	0.0000	1.00000	1.00000
c_10	22	0.9091	0.0610	0.78957	1.00000
c_13	22	0.9752	0.0279	0.92052	1.00000
c_17	22	0.7603	0.1107	0.54337	0.97729
c_21	22	0.9091	0.0610	0.78957	1.00000
c_56	22	0.8678	0.0782	0.71444	1.00000

Classifier with AUC of 1.0: c_4

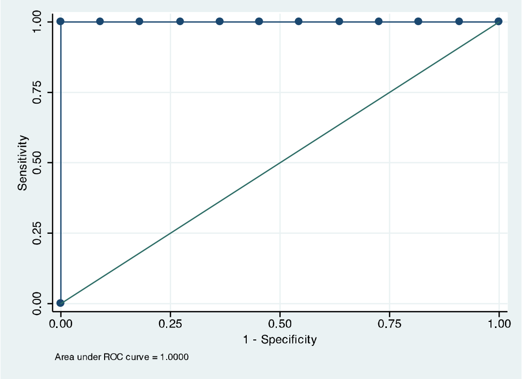

c_4 as classifier

Cutpoint	Sensitivity	Specificity	Correctly Classified	LR+	LR-
(>= .711)	100.00%	0.00%	50.00%	1.0000	
(>= .719)	100.00%	9.09%	54.55%	1.1000	0.0000
(>= .723)	100.00%	18.18%	59.09%	1.2222	0.0000
(>= .755)	100.00%	27.27%	63.64%	1.3750	0.0000
(>= .787)	100.00%	36.36%	68.18%	1.5714	0.0000
(>= .789)	100.00%	45.45%	72.73%	1.8333	0.0000
(>= .795)	100.00%	54.55%	77.27%	2.2000	0.0000
(>= .797)	100.00%	63.64%	81.82%	2.7500	0.0000
(>= .82)	100.00%	72.73%	86.36%	3.6667	0.0000
(>= .829)	100.00%	81.82%	90.91%	5.5000	0.0000
(>= .835)	100.00%	90.91%	95.45%	11.0000	0.0000
(>= .977)	100.00%	100.00%	100.00%		0.0000
(>= 1.003)	90.91%	100.00%	95.45%		0.0909
(>= 1.025)	81.82%	100.00%	90.91%		0.1818
(>= 1.054)	72.73%	100.00%	86.36%		0.2727
(>= 1.076)	63.64%	100.00%	81.82%		0.3636
(>= 1.089)	45.45%	100.00%	72.73%		0.5455
(>= 1.142)	36.36%	100.00%	68.18%		0.6364
(>= 1.162)	27.27%	100.00%	63.64%		0.7273
(>= 1.269)	18.18%	100.00%	59.09%		0.8182
(>= 1.445)	9.09%	100.00%	54.55%		0.9091
(>= 1.481)	0.00%	100.00%	50.00%		1.0000
(> 1.481)	100.00%	0.00%	50.00%	1.0000	
